# HLA class I (-A, -B, -C) and class II (-DR, -DQ) polymorphism in the Mauritanian population

**DOI:** 10.1186/s12881-017-0514-4

**Published:** 2018-01-03

**Authors:** Cheikh Tijani Hamed, Ghlana Meiloud, Fatimetou Veten, Mouna Hadrami, Sidi M. Ghaber, Ely C. Boussaty, Norddine Habti, Ahmed Houmeida

**Affiliations:** 1 Unité de Recherche sur les Biomarqueurs dans la Population Mauritanienne, Université des Sciences de Technologies et de médecine (USTM), Nouakchott, Mauritanie; 2 Laboratoire d’hématologie Faculté de Médecine, Université des Sciences de Technologies et de médecine (USTM), Nouakchott, Mauritanie; 3 Centre National de Transfusion Sanguine, Nouakchott, Mauritanie; 4 Laboratoire d’hématologie et de génie génétique et cellulaire, Faculté de Médecine et de Pharmacie de Casablanca, Université HASSAN II-Ain Chock, Casablanca, Maroc

**Keywords:** HLA, Population, Mauritania, Pcr-ssp

## Abstract

**Background:**

HLA antigens have been widely studied for their role in transplantation biology, human diseases and population diversity. The aim of this study was to provide the first profile of HLA class I and class II alleles in the Mauritanian population.

**Methods:**

HLA typing was carried in 93 healthy Mauritanian blood donors, using single specific primer amplification (PCR-SSP).

**Results:**

Occurrences of the main HLA class I (-A, -B, -C) and class II (-DR, -DQ) antigens in the general population showed that out of the 17 HLA-A allele groups detected, five main HLA-A allele groups: A*02 (18.42%), A*01 (14.04%), A*23 (14.04%), A*30 (13.16%) and A*29 (12.28%) were the most common identified along other 12 relatively minor allele groups. Twenty three allele groups were observed in the locus B of which B*07 (13.46%) was the most prevalent followed by B*15, B*35, B*08 and B*27 all, with a frequency between 7 to 8%. Three prevalent HLA-C allele groups (C*02: 35.09%, C*07: 20.19% and C*06: 13.6%) were detected. The main HLA class II observed allele groups were: DRB1*13 (27.42%), DRB1*03 (24.73%), DRB1*11 (13.98%), DQB1*03 (36.03%), DQB1*02 (22.06%) and DQB1*05 (18.8%). Except for few haplotype in class I (A*02-B*07: 4.45%, A*02-C02: 10%, A*23-C*02: 8.8%, B*07-C*02: 8.8%, B*15-C*02: 8.8%) and in class II (DRB1*13-DQB1*06: 11.94%, DRB1*03-DQB1*02:11.19% and DRB1*03-DQB1*03: 10.45%), the majority of locus combination were in the range of 2–3%. A single predominant haplotype C*02-DRB1*03 (16.67%) was found.

**Conclusions:**

These results, in agreement with previous data using different tissues markers, underlined the ethnic heterogeneity of the Mauritanian population.

## Background

Matching of the two most known human leucocyte antigen groups i.e. HLA class I and class II is the main immunological barrier to organ transplantation. Although these two glycoproteins do not have the same cellular distribution and participate in the antigen peptide presentation to the effector cells in two different ways [1], both molecules segregate in the same codominant inheritance and are highly polymorph [2]. Class I HLA molecules are encoded by three genes termed HLA-A, HLA-B, and HLA-C while six genes (HLA-DPA1, HLA-DPB1, HLA-DQA1, HLA-DQB1, HLA-DRAand HLA-DRB1) encode respectively for the two polypeptides α and β, forming the HLA class II molecules [3]. Despite the astonishing genetic variation of this complex [4], HLA alleles and haplotypes distribution is not fortuitous and their frequencies differ specifically among human populations [5, 6]. Due to the rising interest in HLA typing for hematopoietic stem cell transplantation but also for organ transplantation [7, 8], diseases susceptibility [9] and anthropology purposes [10, 11], efforts to gather databases on this system polymorphism have been undertaken in many countries using, increasingly, reliable molecular technology [12–14].

The present Mauritanian population consists of two ethnic groups: the Moors (Maures) composed of white and black Moors. This group constitutes about 85% of the general Mauritania population and ethnically self-identifies with the neighboring North Africa populations. The second ethic group, the Mauritanian black Africans (15%), consists of three sub ethnic groups (Pulhars, Soninkés and Wolofs) all from the same sub-Saharan black African descent [15].

We have previously investigated the ethnic occurrence of different tissue compatibility determinants such as ABO, rhesus and Kell groups in our population [16, 17]. This study provides the first available source on the global frequencies and ethnic distribution of HLA class I and II antigens in the Mauritanian population.

## Methods

### Cohort description

Subjects of this study were volunteers, from both genders, coming for blood donation at the national center of blood transfusion (CNTS), in Nouakchott, the capital city. Ethnic origin identification of selected individuals was based on first and family names, geographic origin and spoken language. The regulation of the CNTS being that blood from all donors reactive for HIV, Hepatitis B or syphilis should be discarded, positively tested individuals for these infections were therefore excluded from HLA typing.

Eventually, ninety three unrelated and apparently healthy Mauritanians (30 whites Moors, 34 black Moors and 29 Mauritanian black Africans) were recruited. Due to the specific cost of some of HLA typing kits, the number of individuals screened per HLA locus varied. Hoverer, for each gene, typed subjects included the three ethnic groups of our population. This study was approved by the national center of blood transfusion on behalf of the ministry of health and the ethic and research committee of the University of Sciences, Technologies and Medicine (USTM) of Mauritania.

### HLA typing

Peripheral whole blood was collected in EDTA tubes from all selected individuals. Genomic DNA extraction, from buffy coat samples obtained by pretreatment of whole blood, was carried out using Quiagen DNA purification Kit according to the protocol described by the manufacturer. Molecular typing was performed by sequence-specific primer amplification (PCR-SSP) using *One Lambda* DNA generic typing trays pre-coated with primers specifically designed to detect HLA polymorphism. We followed the protocol provided by the manufacturer with the typing kit. Briefly, after the PCR step, electrophoresis on 2.5% agarose and band visualization by a gel imager system (gel doc *VILBER LORMAT*), positive result was indicated by the presence of a specifically amplified DNA fragment as a third band between the internal control product band (conserved region of the Human β globin) and the unincorporated primers band. Absence of specific allele (negative result) was reflected by only two bands (no third band). HLA allele assignment was performed using HLA Fusion™ identification Software.

### Data analysis

HLA allele groups and haplotype frequencies, linkage Disequilibrium, relative linkage disequilibrium (RLD or D’) and Hardy-Weinberg equilibrium (HWE) were performed using the Arlequin suite version 3.5 software (Institute of Ecology and Evolution, University of Bern, Switzerland; http://cmpg.unibe.ch/ [18]. Allele groups and haplotype frequencies were also checked using Excell software.

## Results

### HLA allele groups in the general population

Occurrences of the main HLA class I (−A, -B, -C) and class II (−DR, −DQ) allele groups in the general population (Table 1) showed that out of the 17 HLA-A allele groups detected, A*02 (18.42%) was the most frequent followed sequentially by A*01, A*23, A*30 and A*29 all having a frequency above 12%. The percentage of the twelve remaining allele groups did not exceed 5.5%. A similar pattern was observed in HLA-B with B*07 (13.46%) being the prevalent allele group tailed by B*15, B*35, B*08 and B*27 with frequencies near to 8%. All others allele groups had minor presence. HLA-B showed the widest spectrum with 24 allele groups detected. A relatively reduced number of HLA-C allele groups (13 in total) were identified including C*02 (35.09%), C*07 (20.18%) and C*06 (13.6%). As in class I, this outline was also observed in HLA class II where three major allele groups were identified in HLA-DR (DRB1*13: 27.42%, DRB1*03: 24.73% and DRB1*11: 13.98%) along nine minor allele groups. In contrast, HLA-DQ was the least polymorph locus as only five different allele groups were detected. Besides, differences in frequencies were also less perceptible in this locus compared to the four other loci.

**Table 1:** HLA class I and class II frequencies in the general Mauritanian population

A*alleles	2 *N* = 114	B*allele	2 *N* = 104	C*allele	2 *N* = 114	DRB1*allele	2 *N* = 186	DQB1*allele	2 *N* = 136
	Frequency (%)		Frequency (%)		Frequency (%)		Frequency (%)		Frequency (%)
A*01	14.04	B*07	13.46	C*01	6.14	DRB1*01	2.69	DQB1*02	22.06
A*02	18.42	B*08	7.69	C*02	35.09	DRB1*03	24.73	DQB1*03	36.03
A*03	3.51	B*14	0.69	C*03	6.14	DRB1*04	4.30	DQB1*04	7.35
A*11	0.88	B*15	8.65	C*04	0.88	DRB1*07	8.06	DQB1*05	18.38
A*23	14.04	B*18	3.85	C*05	2.63	DRB1*08	4.84	DQB1*06	16.18
A*24	2.63	B*27	7.69	C*06	13.16	DRB1*09	0.54		
A*26	2.63	B*35	8.65	C*07	20.18	DRB1*10	6.45		
A*29	12.28	B*37	3.85	C*08	3.51	DRB1*11	13.98		
A*30	13.16	B*39	1.92	C*14	1.75	DRB1*12	0.54		
A*32	2.63	B*40	2.88	C*15	0.88	DRB1*13	27.42		
A*33	3.51	B*41	0.69	C*16	5.26	DRB1*14	2.69		
A*34	1.75	B*42	3.85	C*17	3.51	DRB1*15	4.00		
A*36	0.88	B*44	6.73	C*18	0.88				
A*66	1.75	B*45	4.81						
A*69	0.88	B*49	1.92						
A*74	5.26	B*50	5.77						
A*80	1.75	B*51	6.73						
		B*52	1.92						
		B*53	0.69						
		B*56	0.69						
		B*57	0.69						
		B*58	2.88						
		B*78	0.69						
		B*82	0.69						

### Ethnic distribution of HLA alleles

Although most HLA allele groups found in the general population were also present at the ethnic level, differences were observed in the specific order of prevalence of these allele groups (Tables 2 and 3). For instance, HLA-A*01, fourth both in the black Moors (11.9%) and white Moors (10.53%), was the most represented in the Mauritanian black Africans (20.59%) followed by A*02 (14.71%). A*02 was, instead, first both in the black Moors (21.43%) and the white Moors (18.42%). B*07, the most frequent in the white Moors (16.67%), was second (11.11%) in the black Moors and the Mauritanian black Africans (12.5%). HLA-B*27 was second in the black Africans with a noticeably high prevalence (12.50%) compared to its equal occurrence both in the black and whites Moors (5.56%). No signification variation was observed for these allele groups between the different ethnic groups as show by the χ2 and *p* values in, respectively, HLA-A (χ2 = 3.639, 8 ddl; *p* = 0.88), B (χ2 = 10.952, 10 ddl; *p* = 0.36) and C (χ2 = 10.398, 6 ddl; *p* = 0.10).

**Table 2:** Ethnic distribution of class I HLA

HLA-A				HLA-B				HLA-C			
Alleles	WM 2 *N* = 38 Frequency (%)	BM 2 *N* = 42 Frequency (%)	BA 2 *N* = 34 Frequency (%)	Alleles	WM 2 *N* = 36 Frequency (%)	BM 2 *N* = 36 Frequency (%)	BA 2 *N* = 32 Frequency (%)	Alleles	WM 2 *N* = 38 Frequency (%)	BM 2 *N* = 42 Frequency (%)	BA 2 *N* = 34 Frequency (%)
A*01	10.53	11.9	20.59	B*07	16.67	11.11	12.5	C*01		4.76	14.71
A*02	18.42	21.43	14.71	B*08	11.11	8.33	3.13	C*02	42.11	33.33	29.41
A*03	7.89	2.38		B*14		2.78		C*03	5.26	7.14	5.88
A*11	2.63			B*15	2.78	16.67	6.25	C*04		2.38	
A*23	15.79	14.29	11.76	B*18	2.78	5.56	3.13	C*05	2.63	4.76	
A*24	5.26	2.38		B*27	5.56	5.56	12.5	C*06	13.16	9.52	17.65
A*26		4.76	2.94	B*35	8.33	2.78	15.63	C*07	28.95	21.43	8.82
A*29	2.63	19.05	14.71	B*37		2.78	9.38	C*08	5.26		5.88
A*30	18.42	9.52	11.76	B*39	5.56			C*14		2.38	2.94
A*32	2.63	4.76		B*40	5.56	2.78		C*15			2.94
A*33	2.63		8.82	B*41	2.78			C*16		9.52	5.88
A*34	2.63	2.38		B*42	2.78	2.78	6.25	C*17	2.63	2.38	5.88
A*36	2.63			B*44	11.11	5.56	3.13	C*18		2.38	
A*66	2.63	2.38		B*45	5.56	5.56	3.13				
A*69			2.94	B*49			6.25				
A*74	5.26	2.38	8.82	B*50	8.33	8.33					
A*80		2.38	2.94	B*51	5.56	11.11	3.13				
				B*52		2.78	3.13				
				B*53			3.13				
				B*56			3.13				
				B*57		2.78					
				B*58	5.56	2.78					
				B*78			3.13				
				B*82			3.13				

*WM* white Moors, *BM* black Moors, *BA* black Africans

χ2 values: HLA-A (χ2 = 3.639 à 8 ddl; *p* = 0.88), B (χ2 = 10.952 à 10 ddl; *p* = 0.36) and C (χ2 = 10.398 à 6 ddl; *p* = 0.10)

**Table 3:** Ethnic distribution of Class II HLA

HLA-DRB1				HLA-DQB1			
Alleles	WM 2 *N* = 60 Frequency (%)	BM 2 *N* = 68 Frequency (%)	BA 2 *N* = 58 Frequency (%)	Alleles	WM 2 *N* = 52 Frequency (%)	BM 2 *N* = 42 Frequency (%)	BA 2 *N* = 42 Frequency (%)
DRB1*01	1.67	4.41	1.72	DQB1*02	25	16.67	23.81
DRB1*03	26.67	23.53	24.14	DQB1*03	26.92	42.86	40.48
DRB1*04	5	2.94	5.17	DQB1*04	7.69	7.14	7.14
DRB1*07	6.67	8.82	8.62	DQB1*05	17.31	19.05	19.05
DRB1*08	6.67	2.94	5.17	DQB1*06	23.08	14.29	9.52
DRB1*09	1.67						
DRB1*10	5	2.94	12.07				
DRB1*11	13.33	16.18	12.07				
DRB1*12		1.47					
DRB1*13	25	30.88	25.86				
DRB1*14	3.33	4.41					
DRB1*15	5	1.47	5.17				

*WM* white Moors, *BM* black Moors, *BA* black Africans

χ2 values: DR (χ2 = 2.377 à 8 ddl; *p* = 0.96) and DQ (χ2 = 5.625 à 8 ddl; *p* = 0.68)

In HLA class II, the main allele groups identified in the general population (DRB1*13, DRB1*03, DQB1*03 and DQB1*02) were also in the same significant range of prevalence in the three different ethnies. However, DQB1*03 frequency in the black Moors (42.86%) and the black Africans (40.48%) were higher than that found in the whites Moors (26.92%). As in class I loci, we did not observe any deviation for HLA class II allele groups distribution in both DR (χ2 = 2.377, 8 ddl; *p* = 0.96) and DQ (χ2 = 5.625, 8 ddl; *p* = 0.68).

Some HLA-allele groups were exclusively found in one of the ethnic groups. For instance, in class I, A*11, A*36 and B*41 found in the white Moors could not be observed in the black Moors or the black Africans. The allele groups (B*14, B*57, C*04, C*18) and (A*69, B*49) were only identified, respectively, in the black Moors and the Mauritanian black Africans. The presence of these apparently ethny specific allele groups were less common in class II antigens where only DRB1*09 in the white Moors and DRB1*12 in the black Moors were detected.

### Most frequent HLA Haplotypes

Frequencies calculated for the main locus haplotypes, with the respective LD and RLD values, are given in Tables 4 and 5. In class I, HLA-(A-B) haplotypes showed, overall, the lowest occurrence with the maximum frequency observed for A*02-B*07 (4.44%). Higher prevalences were seen in HLA-(A-C) and B-C locus associations with, for instance, A*02-C*02 (10%), A*23-C*02 (8.8%), B*07-C*02 (8.8%) and B*15-C*02(8.8%). Frequencies of 2 locus haplotypes from Class II reached higher level with the associations DRB1*13-DQB1*06 (11.94%), DRB1*03-DQB1*02 (11.19%) and DRB1*03-DQB1*03 (10.45%). Remarkably, with a frequency of 16.67%, the most common haplotype was C*02-DRB1*03, resulting from the combination of loci from class I and class II. Except for these haplotypes, the vast majority of haplotypes had a frequency of about 2 to 3%.

**Table 4:** Most encountered HLA (class I-class II) two loci haplotypes

Haplotypes	HF (%)	LDx100	RLD	Haplotypes	HF (%)	LDx100	RLD
A-B				A-C			
A*02B*07	4.44	1.26	0.12	A*02C*02	10.00	3.18	0.27
A*01B*07	3.33	2.03	0.17	A*23C*02	8.88	2.09	0.23
A*02B*15	3.33	1.22	0.17	A*01C*02	4.44	1.21	0.13
A*23B*07	3.33	0.94	0.08	A*01C*06	4.44	1.66	0.15
A*23B*08	3.33	1.78	0.27	A*02C*06	4.44	1.08	0.1
A*30B*50	3.33	1.14	0.23	A*23C*07	3.33	1.55	0.14
A*01B*37	2.22	1.41	0.42	A*30C*02	3.33	−1.11	−0.24
A*02B*35	2.22	0.26	0.04	A*30C*07	3.33	0.85	0.08
A*02B*39	2.22	1.55	1	A*02C*07	2.22	−1.08	−0.29
A*02B*42	2.22	1.18	0.38	A*03C*07	2.22	1.04	0.37
A*23B*15	2.22	0.68	0.09	A*29C*02	2.22	−2.55	−0.59
A*23B*27	2.22	0.81	0.12	A*29C*07	2.22	0.15	0.02
A*30B*15	2.22	0.75	0.1				
A*30B*27	2.22	0.88	0.13	DRB1-DQB1			
				DRB1*13DQB1*06	11.94	7.04	0.61
B-C				DRB1*03DQB1*02	11.19	5.7	0.35
B*07C*02	8.88	3.86	0.44	DRB1*03DQB1*03	10.45	1.36	0.08
B*15C*02	8.88	4.61	0.83	DRB1*13DQB1*03	8.21	−2.49	−0.23
B*27C*02	5.55	3.03	0.61	DRB1*13DQB1*05	6.72	1.15	0.09
B*35C*02	4.44	0.76	0.14	DRB1*11DQB1*03	5.22	0.9	0.01
B*44C*07	4.44	2.49	0.46	DRB1*07DQB1*02	4.48	3.02	0.57
B*08C*02	3.33	0.15	0.03	DRB1*10DQB1*05	4.48	3.36	0.69
B*37C*06	3.33	2.33	0.71	DRB1*11DQB1*02	3.73	0.18	0.01
B*40C*07	3.33	2.3	1	DRB1*11DQB1*04	2.99	1.76	0.28
B*07C*06	2.22	−0.09	−0.01	DRB1*11DQB1*05	2.99	−0.08	−0.03
B*07C*07	2.22	0.75	0.16	DRB1*08DQB1*03	2.24	1.17	0.61
B*08C*03	2.22	0.15	0.23	DRB1*13DQB1*04	2.24	0.01	0.01
B*08C*07	2.22	1.33	0.22	DRB1*01DQB1*03	1.49	0.69	0.48
B*35C*01	2.22	2.37	0.56	DRB1*03DQB1*05	1.49	−3.25	−0.68
				DRB1*03DQB1*06	1.49	−3.67	−0.64
A-DRB1				DRB1*04DQB1*03	1.49	0.42	0.22
A*02DRB1*03	11.9	4.3	0.32				
A*01DRB1*07	5.95	3.35	0.48	A-DQB1			
A*23DRB1*03	5.95	1.62	0.15	A*30DQB1*03	8.33	2.68	0.33
A*74DRB1*13	5.95	2.81	0.75	A*02DQB1*02	7.14	3.85	0.26
A*23DRB1*13	4.76	−0.99	−0.21	A*02DQB1*03	7.14	−0.44	−0.06
A*30DRB1*13	4.76	1.41	0.16	A*01DQB1*02	5.95	3.06	0.27
A*02DRB1*13	3.57	−2.15	−0.37	A*23DQB1*02	5.95	1.27	0.11
A*29DRB1*03	3.57	1.19	0.12	A*23DQB1*03	4.76	1.15	0.12
A*30DRB1*11	3.57	−0.03	−0.01	A*01DQB1*03	3.57	−0.64	−0.13
A*01DRB1*03	2.38	−1.11	−0.28	A*01DQB1*05	3.57	0.25	0.02
A*01DRB1*11	2.38	−0.29	−0.14	A*02DQB1*05	3.57	−0.5	−0.15
A*03DRB1*13	2.38	0.66	0.26	A*29DQB1*06	3.57	1.34	0.13
A*23DRB1*15	2.38	2.19	0.71	A*74DQB1*03	3.57	0.76	0.22
A*29DRB1*11	2.38	0.87	0.08	A*23DQB1*05	2.38	−0.63	−0.26
A*30DRB1*03	2.38	−1.53	−0.45	A*23DQB1*06	2.38	−0.76	−0.3
A*30DRB1*10	2.38	1.24	0.31	A*29DQB1*04	2.38	1.78	0.28
A*34DRB1*13	2.38	0.33	0.26	A*30DQB1*04	2.38	0.89	0.14
A*66DRB1*13	2.38	1.24	1	A*30DQB1*05	2.38	−0.33	−0.15
				C-DRB1			
B-DRB1				C*02DRB1*03	16.67	4.29	0.25
B*07DRB1*03	5.95	1.51	0.18	C*07DRB1*13	9.52	1.81	0.13
B*15DRB1*03	5.95	2.86	0.49	C*02DRB1*11	4.76	−0.61	−0.12
B*50DRB1*13	4.76	2.08	0.5	C*02DRB1*13	4.76	−4.92	−0.43
B*08DRB1*03	3.57	1.84	0.31	C*03DRB1*13	4.76	1.61	0.37
B*27DRB1*13	3.57	1.44	0.26	C*02DRB1*08	3.57	1.76	1
B*37DRB1*07	3.57	2.64	0.72	C*06DRB1*03	3.57	−0.63	−0.19
B*40DRB1*13	3.57	2.04	1	C*06DRB1*07	3.57	1.69	0.24
B*44DRB1*13	3.57	2.08	0.5	C*06DRB1*13	3.57	0.5	0.06
B*50DRB1*13	3.57	2.08	0.51	C*07DRB1*11	3.57	−0.61	−0.12
B*07DRB1*08	2.38	1.61	0.61	C*08DRB1*13	3.57	2.48	1
B*08DRB1*11	2.38	0.88	0.12	C*02DRB1*10	2.38	0.2	0.07
B*08DRB1*13	2.38	−0.56	−0.21	C*05DRB1*07	2.38	1.59	0.63
B*18DRB1*03	2.38	0.92	0.31	C*06DRB1*11	2.38	−0.94	−0.5
B*35DRB1*03	2.38	−0.09	−0.03	C*07DRB1*03	2.38	−2.54	−0.48

*HF* haplotype frequency, *LD* linkage disequilibrium, *RLD* relative linkage disequilibrium

**Table 5:** Most encountered HLA (class I-class II) 3, 4 and 5 loci haplotypes

Haplotypes	Frequency (%)	Haplotypes	Frequency (%)
A- B -C		A- B -DRB1	
A*02B*07C*02	3.33	A*02B*07DRB1*03	4.76
A*02B*15C*02	3.33	A*01B*37DRB1*07	2.38
A*23B*08C*02	3.33	A*02B*39DRB1*03	2.38
A*01B*07C*02	2.22	A- B- C-DRB1	
A*01B*37C*06	2.22	A*02B*07C*02DRB1*03	3.57
A*02B*35C*02	2.22	A*01B*37C*06DRB1*07	2.38
A*02B*39C*06	2.22	A*02B*39C*06DRB1*03	2.38
A*23B*15C*02	2.22	A*23B*08C*02DRB1*03	2.38
A*23B*27C*02	2.22	A*23B*08DRB1*03	2.38
		A*30B*15DRB1*03	2.38
		A*30B*50DRB1*13	2.38
		A B C DQB1	
		A*02B*07C*02DQB1*02	2.38%
		A*02B*15C*02DQB1*03	2.38%
		A*23B*08C*02DQB1*02	2.38% 2.38%
		A*23B*15C*02DQB1*0	
		A B C DRB1 DQB1	
		A*02B*07C*02DRB1*03DQB1*02	2.38%
		A*23B*08C*02DRB1*03DQB1*02	2.38%
		A*01B*07C*02DRB1*01DQB1*05	1.19%
		A*01B*07C*02DRB1*08DQB1*03	1.19%

## Discussion

In this work, the first carried out in the country, we identified five predominant HLA-A allele groups (A*02, A*01, A*23, A*29 and A*30) in our population. These allele groups were also encountered both in North African [19, 20] and sub-Saharan populations [21, 22]. As in many populations worldwide [10, 23], A*02 was also the most prevalent HLA-A allele group in our cohort. Although A*01 (14.04%), was the second prevalent allele group in our population and among Moroccans and Tunisians [19, 24], its frequency was lower in many sub-Saharan African populations such as in Cameroon (1.1%) [25], black South Africans (2.2%) [22]. or in our southern neighbor Mali (1.7%), [21]. In contrast, the occurrence of A*30 (13.6%) was similar to that detected in sub-Saharan populations: Cameroon (14.26%) [25]. and in Kenya (13.6%) [21]. Frequencies of this allele group were lower in Moroccan (7.7%) [19] and Tunisian (9.4%) [20]. populations. Besides, A*43 a black African specific allele group [23] was not detected in our cohort. This allele was also not found in North African populations [19, 20, 26].

With 24 different allele groups, HLA-B was the most polymorph locus in our study. Subsequently, no allele preponderance was found here. This absence of dominance status was reported in numerous studies [27, 28]. As in HLA-A, we also observed that some of this locus allele groups like B*07 and B*27 had frequencies similar to those in sub-Saharan populations [21]. while the frequencies of allele groups such as B*15, B*35 and B*08, were of the same order as for those observed in the Moroccan and Tunisian populations [19, 24].

Despite the scarcity of data on HLA-C distribution in African populations, similarity in occurrence of some shared alleles (C*02, C*05 and C*18) was also observed between our population and the black South Africans [22].

This resemblance in HLA Class I frequencies between our population and both North African and sub-Saharan populations was also seen in class II antigens as the three main DRB1 alleles detected in this study (DRB1*13, DRB1*03 and DRB1*11) were reported to be common in both populations [19, 21, 22, 24]. DRB1*13, the first ranked HLA-DR allele group in our cohort (27.4.15%) was thus the most common in black South Africans (22%) [22] and in Cameroon (21%) [29]. DRB1*03, the second allele group of this locus in our population (24.73%) was also second both in Moroccan (17.3%) [19]. and Tunisian (20.1%) [24]. populations. Besides, the two most represented DQB1 allele groups in the North African populations (DQB1*03, DQB1*02) were found having the highest frequencies in our cohort. For instance, DQB1*03 percentage (36.03%) was close to that reported in Tunisia (34.03%) [24].

Looking at the distribution of HLA allele groups in the three ethnic entities of the Mauritanian population i.e. the black Moors, the white Moors and the black Africans, we found various examples of frequency resemblance between the white and black Moors on one hand and North African populations on the other hand. For instance, HLA–A*02 frequencies in white Moors (18.4%) and black Moors (21.43%) were comparable to those in the Moroccan (19.1%) [19] and Tunisian (20.9%) [20] populations. This similarity was also observed in the less represented alleles such as DRB1*01, DRB1*08, DRB1*14 or DQ*04 [19, 20].

As the respective proportion of each of the three ethnic groups within the general Mauritanian population was taken into account during the recruitment process, the data provided here could be considered as statistically representative of HLA allele frequencies in our population. These same proportions were used in previous studies [16, 17, 30, 31]. on the distribution of other biomarkers in our country.

Numerous 2, 3, 4 or 5 loci haplotypes were identified in this study, both in class I and class II HLA antigens. However, the main observation was the absence of predominant loci association. This status has been described in various cohorts and was perceived as indication of genetic admixture of the studied populations.

Although no significant ethnic variation was observed on the basis of χ2 and *p* values, our results on the distribution of HLA antigens, coherently with previous studies on other biomarkers [16, 17, 30, 31], suggest genetic differences between the different ethnic groups of the Mauritanian population. The absence of considerable ethnic disparity may be attributed to the limited number of typed individuals in the present cohort and therefore does not reflect, statistically, a real lack of ethnic variation. Sample size has indeed been reported to affect inter-ethnic variation parameters when no methodological implications were to raise [32]. Besides, one of the Hardy–Weinberg Equilibrium requirements is to have a very large population. Consequently, the validity of haplotype frequency estimations from the present study and many others studies carried on populations of limited size is subject to inherent errors such as genetic drift where, for instance, changes in allele frequency may be due to chance. As a result, inferences from the data presented here ought to be considered with caution.

Although molecular HLA typing is very reliable, the cost of this technology may explain why most of screenings carried in our region and worldwide used limited number of individuals. However, bearing in mind the relatively small population of Mauritania (3.5 million inhabitants), the ratio of (typed cohort: general population) in the present cohort is higher than those used in most of studies reporting HLA allele distribution [19, 24, 26, 33, 34].

Still with respect to the sample size, we also concede that the haplotypes frequencies have to also be considered with caution as it is well-known that the frequencies of the most frequent haplotypes are systematically overestimated by the EM algorithm for small sample size. For the same reason, although some HLA alleles were found exclusively in some ethnic groups, given the limited number of typed individuals in each ethnic group, assumption of allele specificity could not be ascertained in this study.

Globally, HLA polymorphism seems to be affected by other less obvious factors which still need to be investigated. Thus, expected similarities in HLA frequencies between populations genetically different and living in regions geographically distant have been reported. For instance, DRB1*11 frequency in Cameroun (7.9%) [21] was closer to that found in China (8.47%) [35] than in the black South African population (20.25%) despite the absence of known geographic or historic connectivity between Cameroon and Chinese populations. Similarly, frequency of DRB1*07 in the Moroccan population (13.9%) [19] had a prevalence closer to that in Kazakhstan (13.1%) [36] than in the neighboring and genetically related Tunisian population (28.6%) [24]. Overlooking the ethnic clustering of the target population during the study design may have contributed in these disparities considering the massive ethnic diversity of human populations. For instance, more than 250 ethnic groups are known to live in Cameroon alone [37].

These two factors i.e. the size of the cohort and the huge ethnic diversity of human populations added to the known high variation of HLA, constitute, to our view, a drawback to general population studies on polymorphic antigens such as HLA. Instead, characterization of these biomarkers in ethnically or geographically restricted groups may be more pertinent, through trans ethnic analysis, in understanding the heritability of population common genetic traits and diseases associated variants.

Although this study was purposely carried out to assess the prevalence and ethnic distribution of major HLA class I and class II markers in our population, we have noticed, in our cohort, a relatively high prevalence of HLA-B*27 particularly in the black Africans (12.5%). Giving the strong and consistent association of this allele with ankylosing spondylitis (AS), a well known inflammatory joint disease [38], the occurrence of this antigen in our population, especially in this group, should be investigated further. The same recommendation may apply to the high frequency of HLA-DRB1*03 found in our cohort as a possible risk factor allele of type 1 diabetes mellitus [39, 40].

Although we agree that HLA high resolution typing must be considered as an important factor in transplantation decisions, in most class I and class II HLA allele, there was no statistical difference on the transplant outcome when allele discrimination is carried out at the low-resolution or high resolution levels [41]. Besides, the number of the HLA alleles list is still growing and many HLA alleles are yet to be identified. As a result, DNA-based low resolution is still accepted in many unrelated allogenic transplantation if high resolution cannot be achieved namely in developing countries. Most of the studies carried out in these populations used first field (2-digit) typing resolution [19, 20, 24, 26, 33, 34] due to the excessive cost of the high resolution.

## Conclusions

These results are coherent with previous data we reported on the ethnic heterogeneity of the Mauritanian population using different tissues antigens. The presence of some human disorders associated HLA alleles in significant prevalence showed the need of further investigation of the role of HLA antigens in disease susceptibly in our population.

The availability for the first time, in our country, of a reliable HLA typing technique is an important step in the development of organ transplantation facilities yet almost inexistent.

## Abbreviations

HLA: Human leucocyte antigen groups
i.e.: In essence
ssp.: Sequence specific primer
